# Letter from the Editor in Chief

**DOI:** 10.19102/icrm.2024.15127

**Published:** 2024-12-15

**Authors:** Devi Nair


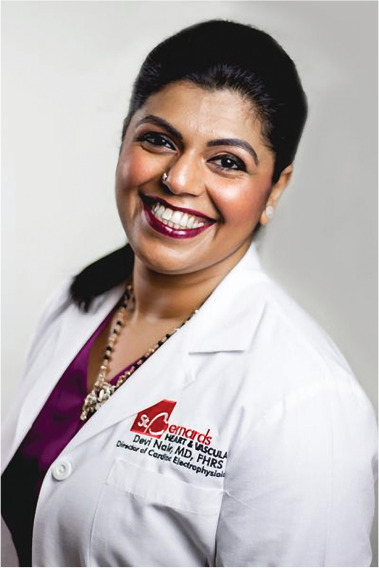


Dear Readers,

As we approach the end of 2024, I am delighted to reflect on what has been a remarkable year for the field of cardiac rhythm management. This year, we have witnessed significant advances across the spectrum of electrophysiology—from groundbreaking trials to transformative technologies—all aimed at improving patient outcomes and refining our understanding of complex arrhythmias. Through the pages of this journal, we have shared and celebrated many of these milestones, fostering a global dialogue that has pushed the boundaries of innovation.

## Highlights of 2024: A Year of Discovery and Progress

In 2024, pulsed field ablation (PFA) solidified its position as a transformative technology in atrial fibrillation (AF) ablation. Early in the year, pivotal trials like *admIRE* and *SPHERE Per-AF* demonstrated the safety and efficacy of PFA systems, paving the way for broader adoption worldwide. By November, the approval of two new PFA systems by the U.S. Food and Drug Administration, in addition to the already approved two systems, further underscored its clinical value, offering precision, tissue selectivity, and safety advantages over traditional thermal ablation. The rapid adoption of PFA across electrophysiology centers marks a critical shift toward more tailored and efficient therapies.

In conduction system pacing (CSP), the *International Collaborative Left Bundle Branch Area Pacing Study (I-CLAS)*, presented at the 2024 Asia Pacific Heart Rhythm Society conference, highlighted the potential of CSP as a new standard for cardiac resynchronization therapy. This study reported reduced hospitalizations and mortality rates associated with CSP compared to biventricular pacing, especially in patients with left bundle branch block.

We also celebrated significant advances in leadless pacing and integrated therapies. Rapid adoption and publications on leadless strategies, both single- and dual-chamber and modular approaches, along with a new DRG code enabling combined AF ablation and left atrial appendage closure procedures, underscored the increasing focus on comprehensive, patient-centered care.

## December 2024 Issue Highlights

This month, we are proud to feature the top three abstracts from the *2024 International Physiology of Pacing Symposium*. These abstracts, selected for their scientific rigor and clinical impact, reflect the symposium’s mission to deepen our understanding of pacing physiology. Accompanying these abstracts is an introduction from the program committee, contextualizing the presented research and its implications for the field.

The December 2024 issue is a testament to our commitment to showcasing excellence and innovation. It captures the spirit of collaboration and inquiry that has defined 2024, while setting the stage for even greater achievements in the coming year.

## Looking Ahead to 2025

As we welcome 2025, the horizon is filled with promise. Emerging technologies, such as expanded applications of PFA, new-generation CSP devices, and integrative therapeutic strategies, hold the potential to revolutionize our practice. Equally exciting are the questions that remain—questions about optimizing treatment protocols, enhancing procedural safety, and broadening access to advanced therapies for diverse patient populations.

Our journal will continue to serve as a platform for addressing these challenges, sharing critical discoveries, and fostering global collaboration. Together, we will work to translate innovation into improved patient outcomes and a deeper understanding of cardiac rhythm management.

## A Note of Gratitude

I extend my heartfelt thanks to our authors, reviewers, and readers for their contributions and support throughout this extraordinary year. Your dedication and expertise have been instrumental in advancing the journal’s mission and enriching our shared field. As we move into the new year, I look forward to continuing this journey with you, exploring new horizons in cardiac electrophysiology.

Wishing you a joyful holiday season and a prosperous New Year!

Warm regards,


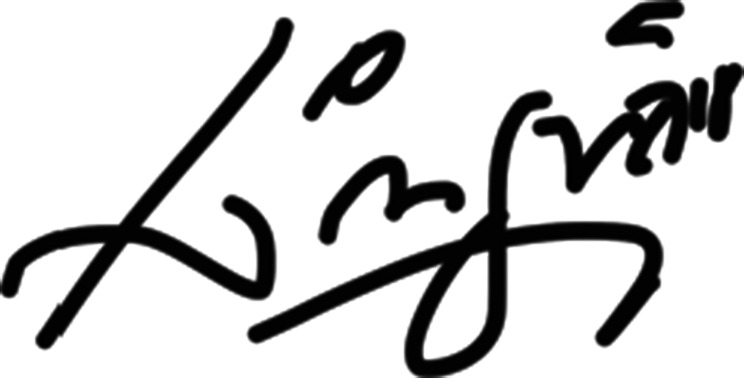


Dr. Devi Nair, md, facc, fhrs

Editor-in-Chief

*The Journal of Innovations in Cardiac Rhythm Management*

Director of the Cardiac Electrophysiology & Research,

St. Bernard’s Heart & Vascular Center, Jonesboro, AR, USA

White River Medical Center, Batesville, AR, USA

President/CEO, Arrhythmia Research Group

Clinical Adjunct Professor, University of Arkansas for Medical Sciences

Governor, Arkansas Chapter of American College of Cardiology

drdgnair@gmail.com

